# Supplementary material for: Modeling glioblastoma heterogeneity as a dynamic network of cell states
Source: Mol Syst Biol. 2021 Sep 16;17(9):e10105. doi: 10.15252/msb.202010105 (PMC8444284; doi:10.15252/msb.202010105)
Supplement: Supplementary file 6 — Source Data for Figure 5 [file MSB-17-e10105-s004.zip › Figure5A_sourcedata/GSEA_3017/nosigdb_stateE.GseaPreranked.1622795031093/VANGRONINGEN_MES.html]

Details for gene set VANGRONINGEN\_MES[GSEA]

|  || Dataset | state63017 |
| Phenotype | NoPhenotypeAvailable |
| Upregulated in class | na\_pos |
| GeneSet | VANGRONINGEN\_MES |
| Enrichment Score (ES) | 0.57513547 |
| Normalized Enrichment Score (NES) | 3.8271108 |
| Nominal p-value | 0.0 |
| FDR q-value | 0.0 |
| FWER p-Value | 0.0 |
Table: GSEA Results Summary

  

Fig 1: Enrichment plot: VANGRONINGEN\_MES      
 Profile of the Running ES Score & Positions of GeneSet Members on the Rank Ordered List

  

| PROBE | GENE SYMBOL | GENE\_TITLE | RANK IN GENE LIST | RANK METRIC SCORE | RUNNING ES | CORE ENRICHMENT || 1 | CYR61 |  |  | 3 | 1.169 | 0.0394 | Yes |
| 2 | IGFBP5 |  |  | 4 | 1.015 | 0.0765 | Yes |
| 3 | ID3 |  |  | 6 | 0.972 | 0.1109 | Yes |
| 4 | SPARCL1 |  |  | 7 | 0.963 | 0.1461 | Yes |
| 5 | ANXA1 |  |  | 12 | 0.826 | 0.1718 | Yes |
| 6 | CD44 |  |  | 14 | 0.806 | 0.2002 | Yes |
| 7 | ANXA2 |  |  | 18 | 0.733 | 0.2236 | Yes |
| 8 | CNN3 |  |  | 24 | 0.702 | 0.2436 | Yes |
| 9 | IFITM3 |  |  | 26 | 0.682 | 0.2674 | Yes |
| 10 | GJA1 |  |  | 33 | 0.618 | 0.2833 | Yes |
| 11 | RAB31 |  |  | 35 | 0.598 | 0.3040 | Yes |
| 12 | EMP1 |  |  | 40 | 0.584 | 0.3209 | Yes |
| 13 | CALD1 |  |  | 41 | 0.580 | 0.3421 | Yes |
| 14 | ID1 |  |  | 45 | 0.542 | 0.3585 | Yes |
| 15 | IFITM2 |  |  | 46 | 0.536 | 0.3781 | Yes |
| 16 | MRC2 |  |  | 56 | 0.505 | 0.3864 | Yes |
| 17 | MMP2 |  |  | 57 | 0.503 | 0.4048 | Yes |
| 18 | WLS |  |  | 65 | 0.472 | 0.4142 | Yes |
| 19 | HSPA5 |  |  | 73 | 0.449 | 0.4227 | Yes |
| 20 | COL5A2 |  |  | 83 | 0.434 | 0.4284 | Yes |
| 21 | CD59 |  |  | 89 | 0.418 | 0.4381 | Yes |
| 22 | NES |  |  | 92 | 0.414 | 0.4509 | Yes |
| 23 | SHROOM3 |  |  | 95 | 0.410 | 0.4637 | Yes |
| 24 | TPM1 |  |  | 108 | 0.388 | 0.4644 | Yes |
| 25 | LGALS1 |  |  | 113 | 0.368 | 0.4733 | Yes |
| 26 | FNDC3B |  |  | 123 | 0.354 | 0.4761 | Yes |
| 27 | ERBIN |  |  | 124 | 0.353 | 0.4890 | Yes |
| 28 | CETN2 |  |  | 145 | 0.338 | 0.4788 | Yes |
| 29 | FZD7 |  |  | 164 | 0.323 | 0.4703 | Yes |
| 30 | FILIP1L |  |  | 167 | 0.320 | 0.4797 | Yes |
| 31 | ATXN1 |  |  | 174 | 0.315 | 0.4845 | Yes |
| 32 | LRRC17 |  |  | 182 | 0.310 | 0.4879 | Yes |
| 33 | A2M |  |  | 184 | 0.309 | 0.4981 | Yes |
| 34 | FLNA |  |  | 197 | 0.298 | 0.4954 | Yes |
| 35 | DKK3 |  |  | 205 | 0.293 | 0.4982 | Yes |
| 36 | GNG12 |  |  | 212 | 0.287 | 0.5020 | Yes |
| 37 | MEST |  |  | 215 | 0.285 | 0.5101 | Yes |
| 38 | SDC2 |  |  | 226 | 0.280 | 0.5091 | Yes |
| 39 | PRRX1 |  |  | 229 | 0.278 | 0.5170 | Yes |
| 40 | TMED9 |  |  | 234 | 0.272 | 0.5225 | Yes |
| 41 | EHD2 |  |  | 240 | 0.267 | 0.5266 | Yes |
| 42 | QKI |  |  | 241 | 0.267 | 0.5364 | Yes |
| 43 | CKAP4 |  |  | 250 | 0.262 | 0.5369 | Yes |
| 44 | INSIG1 |  |  | 252 | 0.261 | 0.5453 | Yes |
| 45 | TRAM2 |  |  | 256 | 0.259 | 0.5514 | Yes |
| 46 | LEPROT |  |  | 259 | 0.259 | 0.5586 | Yes |
| 47 | TNFRSF12A |  |  | 262 | 0.257 | 0.5658 | Yes |
| 48 | WWTR1 |  |  | 263 | 0.256 | 0.5751 | Yes |
| 49 | ZFP36L1 |  |  | 287 | -0.255 | 0.5585 | No |
| 50 | ADGRG6 |  |  | 387 | -0.277 | 0.4569 | No |
| 51 | EGFR |  |  | 427 | -0.291 | 0.4235 | No |
| 52 | SEPT10 |  |  | 431 | -0.293 | 0.4309 | No |
| 53 | HOMER1 |  |  | 463 | -0.304 | 0.4070 | No |
| 54 | PLEKHH2 |  |  | 473 | -0.307 | 0.4081 | No |
| 55 | CBFB |  |  | 500 | -0.314 | 0.3902 | No |
| 56 | GDF15 |  |  | 575 | -0.347 | 0.3194 | No |
| 57 | SERPINE2 |  |  | 585 | -0.351 | 0.3220 | No |
| 58 | EGR1 |  |  | 656 | -0.398 | 0.2576 | No |
| 59 | PHTF2 |  |  | 775 | -0.523 | 0.1435 | No |
| 60 | TM4SF1 |  |  | 823 | -0.619 | 0.1131 | No |
| 61 | GAS1 |  |  | 839 | -0.671 | 0.1208 | No |
Table: GSEA details [plain text format]

  

Fig 2: VANGRONINGEN\_MES: Random ES distribution      
 Gene set null distribution of ES for **VANGRONINGEN\_MES**
